# ATP, the ^31^P Spectral Modulus, and Metabolism

**DOI:** 10.3390/metabo14080456

**Published:** 2024-08-18

**Authors:** Jack V. Greiner, Thomas Glonek

**Affiliations:** 1 Department of Ophthalmology, Harvard Medical School, Boston, MA 02115, USA; 2 Schepens Eye Research Institute of Massachusetts Eye and Ear Infirmary, Boston, MA 02114, USA; 3 Department of Ophthalmology, Tufts University School of Medicine, Boston, MA 02114, USA; 4 Clinical Eye Research of Boston, Boston, MA 02114, USA; tglonek@rcn.com; 5 Magnetic Resonance Laboratory, Chicago College of Osteopathic Medicine, Chicago, IL 60615, USA

**Keywords:** ATP, hydrotrope, lens, protein aggregation, ^31^P nuclear magnetic resonance, ^31^P spectral modulus

## Abstract

Adenosine triphosphate (ATP) has a high intracellular millimolar concentration (*ca*. 2.4 mM) throughout the phylogenetic spectrum of eukaryotes, archaea, and prokaryotes. In addition, the function of ATP as a hydrotrope in the prevention of protein aggregation and maintenance of protein solubilization is essential to cellular, tissue, and organ homeostasis. The ^31^P spectral modulus (PSM) is a measure of the health status of cell, tissue, and organ systems, as well as of ATP, and it is based on in vivo ^31^P nuclear magnetic resonance (^31^P NMR) spectra. The PSM is calculated by dividing the area of the ^31^P NMR integral curve representing the high-energy phosphates by that of the low-energy phosphates. Unlike the difficulties encountered in measuring organophosphates such as ATP or any other phosphorylated metabolites in a conventional ^31^P NMR spectrum or in processed tissue samples, in vivo PSM measurements are possible with NMR surface-coil technology. The PSM does not rely on the resolution of individual metabolite signals but uses the total area derived from each of the NMR integral curves of the above-described spectral regions. Calculation is based on a simple ratio of the high- and low-energy phosphate bands, which are conveniently arranged in the high- and low-field portions of the ^31^P NMR spectrum. In practice, there is essentially no signal overlap between these two regions, with the dividing point being *ca*. −3 δ. ATP is the principal contributor to the maintenance of an elevated PSM that is typically observed in healthy systems. The purpose of this study is to demonstrate that (1) in general, the higher the metabolic activity, the higher the ^31^P spectral modulus, and (2) the modulus calculation does not require highly resolved ^31^P spectral signals and thus can even be used with reduced signal-to-noise spectra such as those detected as a result of in vivo analyses or those that may be obtained during a clinical MRI examination. With increasing metabolic stress or maturation of metabolic disease in cells, tissues, or organ systems, the PSM index declines; alternatively, with decreasing stress or resolution of disease states, the PSM increases. The PSM can serve to monitor normal homeostasis as a diagnostic tool and may be used to monitor disease processes with and without interventional treatment.

## 1. Introduction

The relationship of ATP, the ^31^P spectral modulus (PSM) and cell, tissue, and organ energy metabolism begs a historical understanding. The earliest reports of the measurement of the concentration of adenosine triphosphate (ATP) in a living tissue were in the mid-1970s in skeletal muscle [1,2]. These quantitative measurements of the concentrations of ATP in a living tissue were possible with technological advances in phosphorus-31 nuclear magnetic resonance (^31^P NMR) spectroscopy [3]. Quantitative measurements of ATP prior to this were restricted to studies confined to laboratory bench analyses of cellular and tissue homogenates. The concentration of ATP detected by ^31^P NMR in the intact muscle tissue was found to be high, measuring in the millimolar (*ca.* 2.4 mM) range [2]. These measurements in intact living muscle tissue were consistent with the previously reported analytical findings from analyses of homogenates [4]. This high mM concentration of ATP in muscle tissue was anticipated, since muscle is a metabolically active tissue.

Serendipitously, in 1981, we applied this ^31^P NMR technology to the examination of an intact functioning living organ, the crystalline lens [5]. Unexpectedly, we observed and reported the presence of a high millimolar (mM) concentration of ATP. This high (>2.3 mM) concentration of ATP could not be explained in the long-recognized metabolically quiescent lens organ. The observation of a metabolically active muscle tissue vs. a quiescent lens tissue/organ with a similarly high mM concentration of ATP presented a conundrum, and this enigmatic finding led to our study on the high concentration of ATP among living cell, tissue, and organ systems in phylogenetically diverse species [6]. This finding of high mM concentrations of ATP was surprising, and we demonstrated that the high concentration of ATP was inclusive and foundational among eukaryotes, archaea, and prokaryotes. This enigma was perplexing.

ATP is most commonly recognized as the currency and primary source of cellular energy [7]. The Michales constant [8] predicts that only a small micromolar (µM) amount of ATP is required for all the known functions of ATP, combined [9] with the exclusion of the most recent hypothesis that ATP also functions as a hydrotrope [9,10]. The following are the currently recognized functions of ATP in biology:

- A molecular carrier of intracellular energy for processes including ion transport, muscle contraction, nerve impulse propagation, substrate phosphorylation, and chemical synthesis;
- The ultimate metabolic source of high-energy phosphate bonds;
  The parent residue giving rise to vitamin dinucleotides and other cofactors; e.g., NADH, FAD, Co-A, etc.);
- A coenzyme;
- An allosteric enzyme regulator for modulating protein activities;
- A substrate for the first stage of protein synthesis;
- A modifier of the intracellular milieu;
- The principal metabolite for cellular energy transduction mechanisms;
- The transport of macromolecules, such as proteins, into and out of cells;
- A phosphorylating agent in phosphate regulation of transmembrane proteins;
- The source of the adenosine nucleoside, one of the four letters of the genetic code;
- A molecule that participates in the signaling of key bioprocesses;
- A transmitter in intercellular purinergic signaling;
- Hypothesized to be an intracellular hydrotrope, maintaining protein solubilization, preventing non-specific protein aggregation.

The µM ATP concentrations utilized by this listing are in contrast to the mM concentrations of ATP measured among living cell, tissue, and organ systems [6].

The intracellular organelle most responsible for production of ATP is the mitochondrion. The tissue with the most densely packed and voluminously sized mitochondria is muscle. In contrast, crystalline lens tissue has an extremely low concentration of mitochondria, and they are small in size relative to muscle tissue. Moreover, during the course of life, the lens undergoes a maturation process involving denucleation [11] with accompanying loss of intracellular organelles, including mitochondria. Why does such a high concentration of ATP exist among diverse species, their organs, tissues, and cells? The presence of such a high mM concentration of ATP with a broad phylogenetic presence suggests this enigma represents a fundamental property of living cells, tissues, and organs [6].

### 1.1. ATP and Interstitial Water

**Figure 1:**
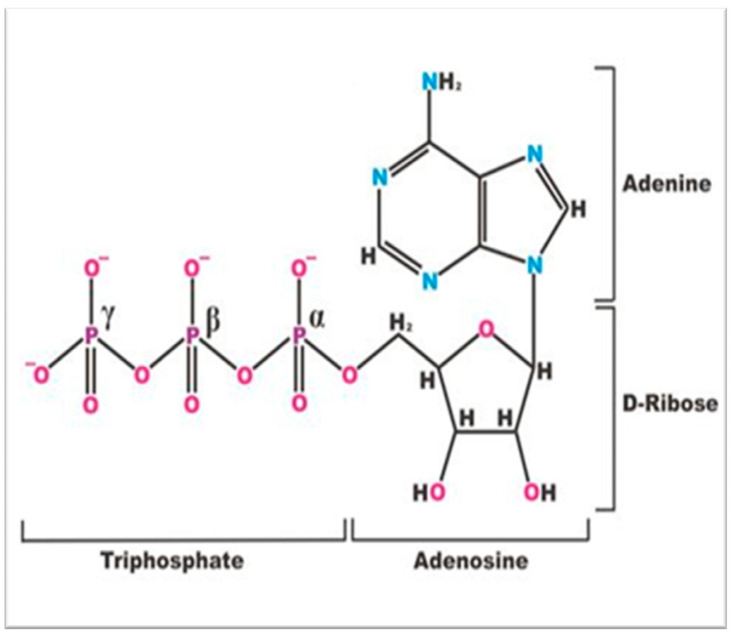
Atomic molecular structure of adenosine triphosphate (ATP) [9] depicting the adenine moiety, the adenosine moiety, comprising purine adenine and the sugar ribose, and the triphosphate residue connected by the sugar ribose. γ, β, and α phosphorus moieties comprising the triphosphate residue are depicted.

After (1) our discovery of the high concentration of ATP in the intact functioning lens organ, and (2) our subsequent studies on the effects of various stressors, such as hypo- [5] and hyperglycemic [12] conditions, glucocorticoids [13], and multiple charged cations [14,15,16] on lens metabolism, we directed our attention to the possible effects that waters-of-hydration have on lens metabolites. More specifically, our focus was directed to the potential interaction of water and the phosphate groups of the ATP tripolyphosphate moiety (Figure 1). 

**Figure 2:**
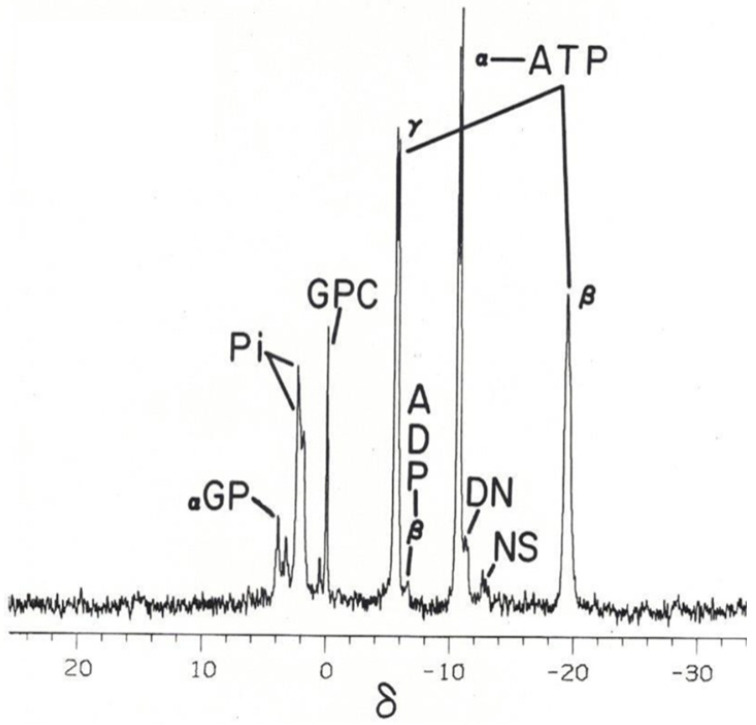
Phosphorus-31 nuclear magnetic resonance (31P NMR) spectrum of an intact Tegu lizard crystalline lens with the γ-, α-, and β-resonance signals of ATP (adenosine triphosphate). The δ (ppm) scale follows the shift convention of the International Union for Pure and Applied Chemistry (IUPAC) and is referenced relative to the resonance position of 85% phosphorus acid [17,18], αGP, alpha-glycerophosphate signal part of the sugar phosphate groups; Pi, inorganic orthophosphate; GPC, glycerolphosphorylcholine; ADP, adenosine diphosphate; DN, the dinucleotides; NS, nucleoside sugar phosphates. The identity of the spectral shift positions assigned to each organophosphate in the above spectrum are recognized in *Methods in Enzymology* [4].

Conveniently, the phosphate groups of ATP broadcast their signals in a magnetic field in such a way that each of the three phosphate groups resonate at different spectral locations when deployed in a ^31^P nuclear magnetic resonance (^31^P NMR) spectrum. As such, each phosphate group denotes their molecular position in ATP’s triphosphate chain: γ, the terminal end-group phosphate, possessing a weak-acid functional group; β, the middle-group phosphate of the triphosphate chain, a strong acid; α, an esterified end-group phosphate, a strong acid (Figure 2). This permits the observation of ATP individual phosphate interactions with the intracellular milieu of interstitial water molecules and solutes. These three phosphates spin-couple to each other, resulting in the doublet, doublet, triplet, ^31^P-^31^P fine-structure splitting of each phosphate’s resonance signal.

### 1.2. Signal Splitting among Phosphate Groups of ATP

**Figure 3:**
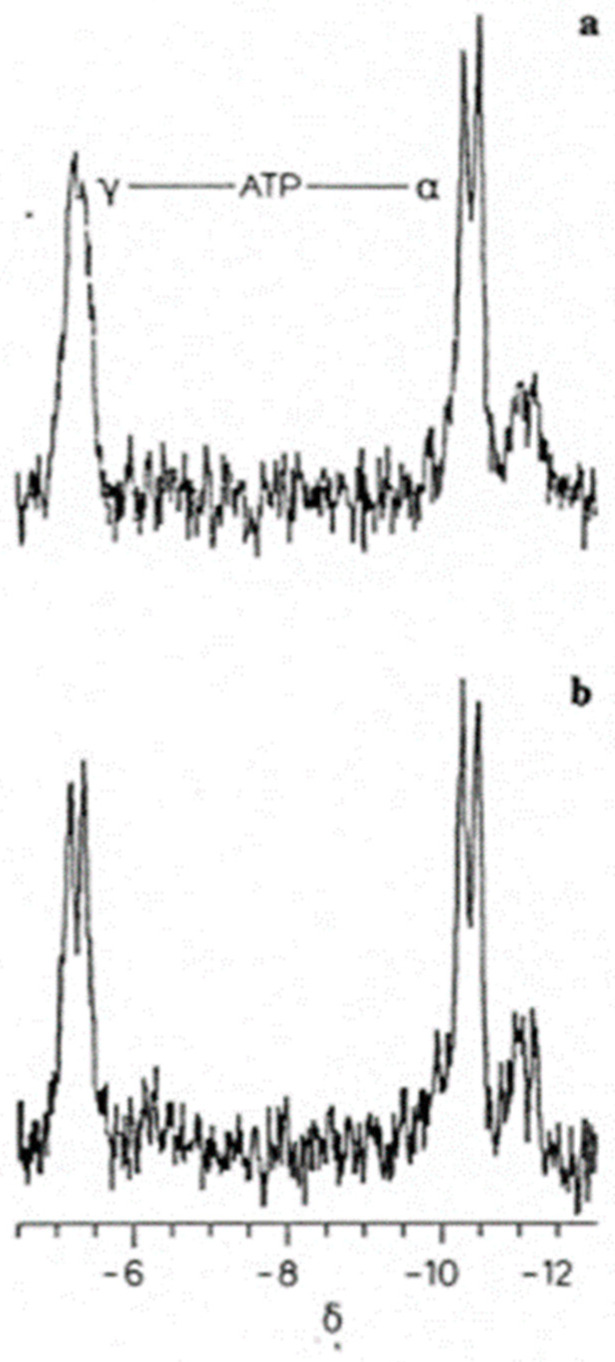
Phosphorus-31 nuclear magnetic resonance (^31^P NMR) spectrum of the γ- and α-group resonance signals of the tripolyphosphate residue of ATP from an ex vivo intact canine crystalline lens [19]. The identity of the spectral shift positions assigned to the ATP γ- and α-group phosphates in the above spectra are recognized in *Methods in Enzymology* [4]. The signal splitting phenomenon with doublet formation, as illustrated above, has been well described [19]. (**a**) Control demonstrates broadened phosphate signal lines of the ATP γ-group doublet due to rapid proton exchange of this weak-acid ATP phosphate group with the protons of the surrounding interstitial water solvent. (**b**) Ex vivo ^31^P NMR spectrum from an intact canine lens incubated for 3.2 h in D_2_O, with the D_2_O-narrowed signal lines of the ATP γ-group doublet resulting from exchange substitution of the ATP γ-group protons associated with the surrounding hydrogen atoms of the water molecules with the deuterium atom now present in the interstitial water solvent. The γ-phosphate signal narrows because, unlike the protons of the water molecule, the deuterium nucleus spin-couples poorly with the ^31^P phosphate nucleus. Note that both the control and deuterium-incubated spectra were obtained using the same lens and identical NMR instrumental settings. Filter time constant used introduced 1 Hz line broadening to the spectra. Ordinarily, filter time constants introducing 10 Hz or greater line broadening are used in intact-tissue ^31^P spectra. Such filtering usually masks the fine structure of ATP [21].

Incubating the crystalline lens in heavy water permitted us to specifically study the effects of interstitial water on ATP. We tested lens incubation techniques validating the metabolic stability and maintaining metabolic health over a time-course [5,12,13,14,15,16,19]. We incubated the lens in heavy water (deuterium dioxide, D_2_O), using ^31^P NMR intact tissue technology to monitor its effects on ATP. Lens incubation in D_2_O over a time-course of 3.2 h yielded the finding of an unusual influence of water on the phosphate spectral signals of ATP (Figure 3) [20]. During incubation, some of the ^31^P NMR spectral signals denoting the phosphate groups showed signs of increasing signal-width narrowing that revealed the signal’s fine-structure. Both the α- and γ-phosphate signal lines narrowed considerably. This phenomenon occurred most prominently in the terminal γ-group of ATP’s polyphosphate chain (Figure 3) [20]. Briefly, the surrounding solvent D_2_O-narrowed signal lines of the ATP γ-group phosphate doublet. The appearance of the doublet occurred as a result of substitutional exchange of the deuterium atom in place of the water’s usual hydrogen (proton) atom. The γ-group phosphate signal narrows because, unlike the protons of the water molecule, the deuterium nucleus spin-couples poorly with the ^31^P nucleus of the phosphate molecule. The ATP multiplets arise because of ^31^P-^31^P spin coupling between the γ- and β- and the β- and α-phosphate groups [20].

### 1.3. ATP as a Hydrotrope

According to our hypothesis based on ^31^P NNR findings [9], the γ-group phosphate located farthest from the protein-bound ATP adenine and ribose sugar moieties (Figure 1) is least encumbered or influenced by the organization of the intracellular interstitial water molecules. These water molecules are organized at the protein surfaces [20]. The more distal the location of the phosphate group from the adenosine moiety, the less organized and the more fluid are the surrounding water molecules [20]. Using heavy water (D_2_O), we demonstrated that the influence of the organization of interstitial water molecules was least at the terminal γ-group phosphate of ATP (Figure 3). This is where water molecules are less well organized (least ice-like) and thus more fluid. This observation is less likely to be demonstrated in a study of cellular and tissue homogenates [10], where the relationship with water is less apt to be influenced by the organization of intracellular water by, for example, lens crystalline proteins. Extracts of cells and tissue homogenates yield biochemical information from disrupted living cells and tissues where the effect of the organization of water on the triphosphate moiety of ATP is absent. This is a decisive advantage offered when analyzing intact tissue or an intact organ with an exceedingly high concentration of extremely well-organized proteins, such as the crystalline lens [20].

### 1.4. The Hydromolecular Influence of ATP’s Triphosphate

**Figure 4:**
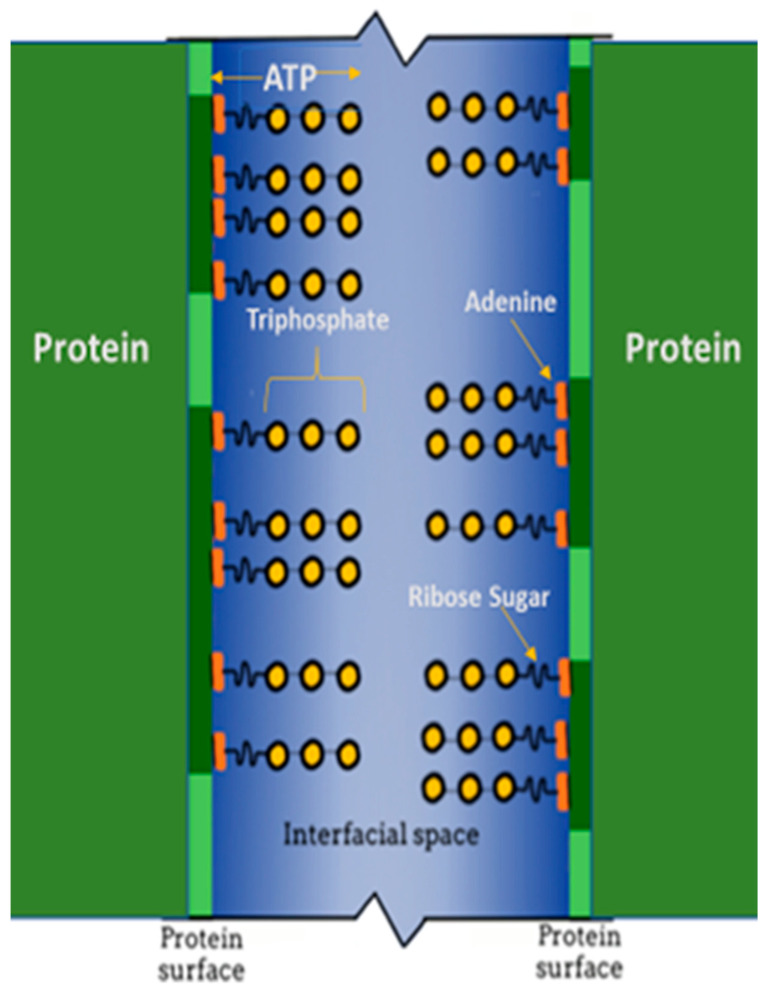
Schematic representation depicting the relationship between adjacent intracellular protein molecules, hydrotropic ATP molecules, and the interfacial space between the protein surfaces [9]. Interstitial water fills the interfacial space (blue), bounded by two adjacent proteins (vertical green lines). The hydrophobic regions of the adjacent proteins (dark green portions of the vertical green lines) interact with the ATP adenine residues (orange). The ATP ribose sugar residue (short wavy line) connects the hydrophobic adenine moiety with the hydrophilic ATP α-, β-, and γ-triphosphate residues (yellow circles). The triphosphate chain residue extends into the interfacial space.

Though we recognized and reported the hydromolecular influence on the most distal phosphate of ATP in 1990 [20], we did not fully appreciate the implication of this until the report of Patel et al. in 2017 [10], nearly three decades later. The postulation of Patel and colleagues, studying cellular and tissue homogenates of cellular and tissue extracts [10], supports our hypothesis (Figure 4) [9]. Our discovery of the split signal spectral peaks relied on our observations in experiments performed on the intact living lens organ, whereas Patel and colleagues reported data obtained from the more disrupted extracts from cell homogenates. The discovery of signal splitting is phenomenologically an example of the importance of the capacity to study ATP in intact living cell, tissue, and organ systems afforded by the advancements in ^31^P NMR technology for studying living tissues [1,2]. The close relationship between organophosphates of the ^31^P NMR spectral profile in intact tissues/organs and tissue and cellular extracts has long been established in our laboratories [3,5,22]. Of even greater importance is the fact that there now exists the ability to study in vivo intact tissues and organs via the ^31^P spectral modulus (PSM) [23].

### 1.5. Hydrotropism

We observed that the α- and γ- phosphate groups of ATP’s triphosphate, as well as their spectral signals, are influenced by surrounding interstitial water molecules [20]. This is a form of hydrotropism. The water molecules influence the ^31^P signals of the phosphate molecules. It is the ordering of the surrounding interstitial neighboring water molecules of the intracellular milieu that influences the NMR signals broadcasted by the ATP phosphate molecules. The influence of the more ordered (ice-like) water molecules is highest the closer the phosphate molecule is to the ribose sugar connecting the triphosphate chain to ATP’s adenine molecule (Figure 4). As we hypothesized, the adenine molecule cloaks the hydrophobic portions of the intracellular proteins [9]. This is especially important in the crystalline lens, since the lens organ possesses a greater protein concentration than any organ in the body. Additionally, the proteins are highly organized.

### 1.6. Lens Protein Organization

Since our model predicts that ATP molecules are oriented with their adenine moiety cloaking the hydrophobic portions of the intracellular proteins, the triphosphate side chains would project into the interstitial space (Figure 4). The interprotein space is filled with interstitial water. As such, the phosphate side chains are surrounded with water. It is this interaction with water molecules that organizes and encapsulates the ATP phosphate residues. As stated above, the most highly ordered water (most ice-like) is at the proximal α-group phosphate. In contrast, the least ordered water is at the γ-group phosphate, where the water molecules are less well organized (least ice-like) and more fluid.

### 1.7. The Lens Model

The crystalline lens presents an opportunity to study an ideal model organ system because it is a self-contained organ entirely encapsulated within a permeable collagenous basement membrane. The lens has no need for a blood supply, since the lens derives its nutritional support by being continuously bathed in oxygenated metabolically rich aqueous humor anteriorly. Moreover, the lens organ can be kept alive and its health status monitored ex vivo via incubation. This allows for organ preservation for extended periods of time, permitting measurement of metabolism over a time-course. This substantiates the metabolic health of the lens organ as measured by maintaining its high concentration of ATP [5].

### 1.8. A Fundamental and Foundational Change

The establishment of ATP with a hydrotropic function in the lens [9] supports a fundamental and foundational change in a long-held hypothesis that the main function of ATP was as the cellular currency. This cellular currency provides readily releasable energy in the bond between the β-group and γ-group phosphates of its triphosphate moiety [7]. The addition of water or hydrolysis to ATP results in the release of this energy with a broad range of cell functions. However, it appears that the more enduring and likely early evolutionarily important function of ATP was that of preventing protein aggregation and maintaining protein solubilization [9,10].

### 1.9. ATP and Protein Aggregation

The function of ATP in preventing protein aggregation is consistent with the suggestion of Patel and colleagues that the use of ATP as the carrier of energy currency was co-opted evolutionarily, having first served to prevent protein aggregation and maintain protein solubilization [10]. The ATP functioning in the prevention of protein aggregation or maintenance of protein solubility is foundational, in that this activity prevents cellular and organ failure. This phenomenon can not only be measured by tissue extracts [10], but ex vivo [9] and in vivo with the ^31^P NMR spectral modulus [23]. Without the high levels of ATP used for energy production and the maintenance of proteins in solution, cell, tissue, and organ hemostasis could not be supported. The decline or absence of this support can result in dysfunction and diseases. With declining ATP concentration resulting in tissue or organ dysfunction and ATP usually being the highest and most dominant of all the metabolites contained in the ^31^P spectrum, a numerically calculated phosphorus-31 spectral modulus [24] is possible. This ^31^P spectral modulus presents a useable and useful index of metabolic health.

### 1.10. ATP and Its Relationship to PSM

The high concentration of ATP and its major roles as (1) the energy currency and (2) to prevent protein aggregation and maintaining protein solubilization, as well as its presence as the dominant organophosphate of the ^31^P NMR tissue spectrum [3,9], serve as a major constituent in the calculation of the ^31^P spectral modulus (PSM). In fact, in healthy cells, tissues, and organs, ATP is the greatest contributor to the ^31^P NMR spectrum [3,6], and, thus, maintains a major prominence in the health maintenance of cellular, tissue, and organ homeostasis.

### 1.11. The ^31^P Spectral Modulus

Considering the above, the ATP-related PSM can be used to measure and monitor the metabolic status of cells, tissues, and organs for initial diagnosis and during the course of treatment. The phosphorus-31 spectral modulus (PSM) can not only be calculated from the ^31^P NMR spectrum of biochemical extracts of cells, tissues, and organs, but also from a ^31^P NMR spectrum of intact or living cells, tissues, and organs. The ^31^P NMR spectrum comprises signals derived from organophosphate metabolites that resonate from 10 δ to −25 δ on the phosphorus chemical shift delta (δ) scale (Figure 2) and needs to be considered in the context of clinical magnetic resonance imaging using surface-coil technology.

The higher the magnetic field strength and the greater the tissue volume, the greater signal-to-noise resolution of signals in the ^31^P spectrum—for example, the difference between the intact tissue and the perchloric acid (PCA) extract [3,5,25]—and using a surface coil permits performing ^31^P NMR on people to acquire useful data. Although as stated above, the PSM can be calculated from the intact or ex vivo tissue, the calculation of the PSM does not require resolution of individual spectral signals. All that is necessary for the PSM calculation in vivo is the spectral integral curve computed from the absolute spectrum of all phosphorus tissue metabolism. It is the integral curve alone, generated by the NMR, that is used to derive a spectral modulus.

### 1.12. The Integral Curve

**Figure 5:**
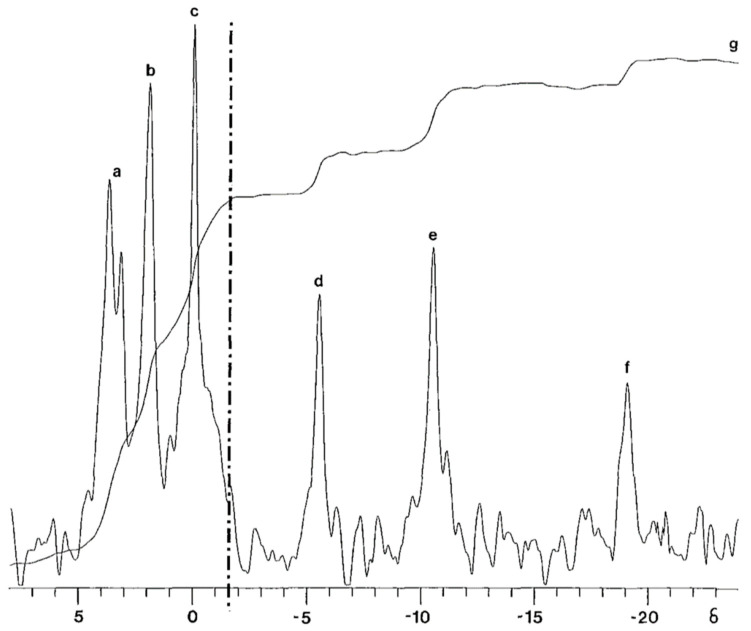
Ex vivo phosphorus-31 nuclear magnetic resonance spectrum and integral curve of a human cornea [26]: (a) sugar phosphate resonance band, (b) inorganic orthophosphate signal, (c) glycerol 3-phosphorylcholine signal, (d) γ-group phosphate resonance of ATP, (e) α-group phosphate resonance of ATP, and (f) β-group phosphate resonance of ATP. The vertical broken line divides low-energy (left) and high-energy spectral regions (right). The quantity of organophosphate beneath the integral curve (g) to the left of the broken line corresponds to the integrated signal area of low-energy phosphates, and the quantity under the integral curve (g) to the right of the vertical broken line corresponds to the integrated signal area of high-energy phosphates. The inflection point is at the intersection of the vertical broken line and the integral curve.

For illustrative purposes herein, the integral curve is superimposed over the ^31^P spectrum (Figure 5) [26]. In the healthy non-diseased cell, tissue, or organ systems, the integral curve corresponding to the down-field or low-field (left-side) portion of the ^31^P NMR spectrum is depressed relative to the up-field or high-field (right side) portion of the ^31^P NMR spectrum. On the up-field end of the spectrum the integral curve is elevated. Between the left (low-field) depressed portion of the curve and the right (high-field) elevated portion of the curve is an intervening region connecting these two portions. In this intervening region of the integral curve is an inflection point (Figure 5). The inflection point is defined as the flattened (horizontal) portion of the intervening region. In the NMR spectrum, the inflection point is located just to the left of the signal representing phosphocreatine at approximately −3 δ. The determination of this inflection point is important for the calculation of the ^31^P spectral modulus (PSM), as it is the point that divides the full spectrum into the low-energy and high-energy spectral sections.

### 1.13. Calculation of the ^31^P Spectral Modulus

The PSM (also known as the energy modulus [24]) is the ratio of high-energy phosphate to low-energy phosphate spectral integrals that may be conveniently grouped into respective spectral sections. These spectral sections are defined by ^31^P chemical shifts of −0.13 δ to 25 δ (ppm) for the high-energy phosphates and 10 to −0.13 δ for the low-energy phosphates. High-energy phosphates are typically described as providing the energy necessary for the oxidative phosphorylation activity of cellular metabolism. We have demonstrated (1) in general, the higher the metabolic activity, the higher the spectral modulus, and (2) the modulus calculation does not require highly resolved ^31^P spectral signals [9,23]. In stressed and diseased tissues ultimately undergoing metabolic decline, the PSM has been shown to be typically low or low relative to a particular tissue [27] or following time-course measurements in dynamic decline [28,29]. We hypothesize that the PSM can be used to differentiate between tissues and organs that are healthy (normal), stressed, and diseased.

## 2. Methods 

In order to determine if the ^31^P spectral modulus could be applied to NMR spectra derived from cells, tissues, or organs from healthy (normal), stressed, and diseased systems, we chose to evaluate established data in the literature. In order to do this, our first and foremost consideration was to control the wide degree of variations found in published data. We entered the following into the manuscript text. In reviewing the past 50 years of the ^31^P spectral NMR literature since the initial presentation of intact living tissue analyses in the mid-1970s required only cases which included full spectral analysis where a catalog of high-energy phosphates and low-energy phosphates was included. This strict criterion severely limited the possible inclusion of findings from the literature to date. This is because there are relatively few studies that provide complete ^31^P spectral data and thus few that could be analyzed for this study. Further compounding the difficulty in obtaining ^31^P spectral information was the high degree of variability from laboratory to laboratory.

Our literature observations of variations in the ^31^P spectral moduli among tissues and organ systems, as well as variations in ATP and moduli reported from laboratory to laboratory [6], indicated that, for analytical precision, this analysis would best be restricted to work performed in our laboratories, where sampling, preparation, and instrumental methods were controlled. The range of variations among laboratories in determinations of ATP concentrations must also include consideration of the quality, sophistication, and precision exercised by the training and level of expertise of investigators. We have previously documented the significant errors that can occur when using different extraction preparations [6], e.g., ATP molarity measured in brain tissue in two comparable reports [30,31], both using the luciferin–luciferase assay for ATP molarity. The first study determined the ATP molarity of brain to be 5.9 mM and the second study to be 121 µM. These values differ by over two orders of magnitude, a finding that raises issues with the accuracy of the luciferin–luciferase assay as applied to biological specimens. As we presented previously, it appears that strong acid extraction of tissue ATP is ordinarily required to obtain the highest tissue ATP molarities [6], a measure of tissue metabolic integrity. The utilization of strong acid extraction by perchloric acid has been validated by ^31^P NMR spectroscopy of intact living tissues, which compared well to tissues prepared by PCA extracts [3,5]. The literature selection herein referenced to our laboratories served to maximize the fidelity of the high-energy and low-energy integral curves used to calculate the ^31^P spectral moduli. The PSM was calculated among healthy (normal), stressed, and diseased systems (respectively, Table 1, Table 2 and Table 3).

Table 1, Table 2 and Table 3 comprise a set of similar data. The tables are organized in an identical format as follows: Column 1 (left) identifies the species examined for each case along with reference to the work from which the data were retrieved. It should be noted that there are significant interspecies differences in phosphatic metabolite profiles, even among the same tissues, for example, the crystalline lens [32] and the cornea [33]. Column 2 describes the nature of the gross sample, whether (1) cells, (2) tissues, or (3) organs. Column 3 describes the nature of sample preparation for NMR analysis, whether (1) in vivo, (2) ex vivo, or (3) a tissue perchloric acid extract (PCA). Column 4 describes the nominal physiological state of the sample before sample preparation, whether (1) healthy (normal), (2) stressed, or (3) diseased. This column provides the variable used to sort the data sets into their appropriate tables for statistical analysis. Columns 5 and 6 present, respectively, the relative high-energy phosphate and low-energy phosphate amplitude for each case. Column 7 presents the computed ^31^P spectral modulus (PSM). The final Column 8 identifies the item in the cited paper from which the data were derived or taken.

Statistical analysis was conducted on the data accumulated in Column 4 in Table 1, Table 2 and Table 3 and included the determination of means and standard deviations and probability testing utilizing the T-test and two-tailed, two-sample unequal variance.

**Table 1:** ^31^P spectral moduli calculated by dividing the high-energy phosphates by the low-energy phosphates from tissues and organs considered normal/healthy and examining them using phosphorus-31 nuclear magnetic resonance (^31^P NMR).

Species (Tissues or Organs) ^Reference^	Nature of Gross Sample (2, Tissues; 3, Organs)	Preparation (1, In Vivo; 2, Ex Vivo; 3, PCA)	Physiological State (1, Normal; 2, Stressed)	High-Energy Amplitude (Relative)	Low-Energy Amplitude (Relative)	^31^P Spectral Modulus (High-Energy)/(Low-Energy)	Source
rabbit lens (intact) [5]	3	2	1	68.4	31.6	2.16	Table 1
rabbit lens (freshly excised) [5]	3	3	1	71.9	28.1	2.56	Table 1
rabbit lens (incub 24 h) [5]	3	3	1	75.8	24.2	3.13	Table 1
rat lens (inact) [25]	3	2	1	52.4	49.0	1.07	Table
rat lens [25]	3	3	1	52.4	49.4	1.06	Table
rabbit lens (freshly excised) [12]	3	3	1	71.9	28.1	2.56	Table II
rabbit lens (incub 24 h) [12]	3	3	1	75.8	29.2	2.60	Table II
rabbit lens (incub fructose) [12]	3	3	1	72.8	27.2	2.68	Table II
rabbit lens (incub 24 h) [13]	3	3	1	75.1	24.2	3.10	Table I
rabbit cornea (intact) [29]	2	2	1	55.6	44.2	1.26	Table I
rabbit cornea (intact) [29]	2	3	1	57.8	42.5	1.36	Table I
human cornea (intact) [34]	2	2	1	53.8	46.2	1.16	Table
rabbit lens (control) [19]	3	3	1	69.0	31.0	2.23	Table II
porcine cornea (intact) [35]	2	2	1	47.3	52.7	0.90	Table 1
bovine cornea (intact) [35]	2	2	1	63.6	36.4	1.75	Table 1
human cornea (intact) [35]	2	2	1	53.8	46.2	1.16	Table 1
human cornea (intact) [26]	2	2	1	n.g.	n.g.	0.75	Table 1
human cornea (intact) [26]	2	2	1	n.g.	n.g.	0.85	Table 1
human cornea (intact) [26]	2	2	1	n.g.	n.g.	1.08	Table 1
human cornea (intact) [26]	2	2	1	n.g.	n.g.	1.01	Table 1
human cornea (intact) [26]	2	2	1	n.g.	n.g.	0.89	Table 1
human cornea (intact) [26]	2	2	1	n.g.	n.g.	0.94	Table 1
human cornea (intact) [26]	2	2	1	n.g.	n.g.	1.10	Table 1
human cornea (intact) [26]	2	2	1	n.g.	n.g.	1.19	Table 1
human cornea (intact) [26]	2	2	1	n.g.	n.g.	0.81	Table 1
human cornea (intact) [26]	2	2	1	n.g.	n.g.	0.85	Table 1
rabbit lens (control) [14]	3	2	1	68.6	31.4	2.18	Table 1
chicken pectoralis [36]	2	2	1	31	12.2	2.54	Table 1
toad gastrocnemius [36]	2	2	1	21.1	8.6	2.45	Table 1
frog gastrocnemius [36]	2	2	1	33	5.9	5.59	Table 1
human quadriceps [37]	2	2	1	20	12	1.67	Figure 7A
rat heart (perfused) [38]	3	3	1	54.1	10.2	5.3	Table 4
rat liver [39]	2	2	1	25.8	45.2	0.57	Table 4
human lens [40]	3	3	1	521	472	1.1	Table 1
rabbit lens (freshly excised) [40]	3	3	1	474	472	1	Table 1
guinea pig brain [41]	2	3	1	65.94	36.93	1.79	Table 1
human quadriceps [21]	2	2	1	52.9	47.1	1.12	Table 1
guinea pig brain [42]	2	3	1	54.80	44.75	1.22	Table 1
rat heart (perfused) [43]	3	2	1	54.07	21.05	2.57	Table 3
rabbit aorta [44]	2	2	1	46.73	53.27	0.88	Table 1
human colon [45]	2	2	1	46.99	57.45	0.82	Table 1
human breast [46]	2	3	1	45.64	57.45	0.79	Table 1
human tumor (benign) [46]	2	2	2	54.27	51.36	1.06	Table 1

Key: Species tissues or organs^Reference.^; Nature of gross sample: 2, tissues; 3 organs; Preparation: 1, in vivo; 2, ex vivo; 3, perchloric acid (PCA) extract; Physiological state: 1, normal; 2, stressed; high-energy (relative) amplitude; low-energy (relative) amplitude; ^31^P spectral modulus; Source, figure/table. n.g., not given.

**Table 2:** ^31^P spectral moduli calculated by dividing the high-energy phosphates by the low-energy phosphates from stressed tissues and organs and examining them using phosphorus-31 nuclear magnetic resonance (^31^P NMR).

Species (Tissues or Organs) ^Reference^	Nature of Gross Sample: (2, Tissues; 3, Organs)	Preparation: (2, Ex Vivo; 3, PCA)	Physiological State: (2, Stressed)	High-Energy Amplitude (Relative)	Low-Energy Amplitude (Relative)	^31^P Spectral Modulus (High-Energy)/(Low-Energy)	Source
rabbit lens (glucose-depleted) [5]	3	3	2	35.3	64.7	0.546	Table 1
rabbit lens (galactose 24 h) [12]	3	3	2	54.6	45.4	1.20	Table II
rabbit lens (dexameth-asone 24 h) [13]	3	3	2	25.8	74.3	0.347	Table I
rabbit lens (ouabain 14 h) [19]	3	3	2	33.9	66.1	0.513	Table II
cat cornea 1 (intact) [47]	2	2	2	38.3	61.8	0.620	Table 2
cat cornea 2 (intact) [47]	2	2	2	32.7	67.3	0.486	Table 2
cat cornea 3 (intact) [47]	2	2	2	43.4	56.5	0.767	Table 2
cat cornea 4 (intact) [47]	2	2	2	37.0	63.0	0.587	Table 2
rabbit lens (verapamil) 13 h) [16]	3	3	2	59.0	41.0	1.44	Table I
rabbit lens (verapamil) [16]	3	2	2	55.1	44.9	1.23	Table I
cat cornea (transplanted 24 h) [26]	2	2	2	n.g.	n.g.	0.62	Table 1
cat cornea (transplanted 24 h) [26]	2	2	2	n.g.	n.g.	0.49	Table 1
cat cornea (transplanted 24 h) [26]	2	2	2	n.g.	n.g.	0.77	Table 1
cat cornea (transplanted 96 h) [26]	2	2	2	n.g.	n.g.	0.49	Table 1
cat cornea (transplanted 96 h) [26]	2	2	2	n.g.	n.g.	0.61	Table 1
cat cornea (transplanted 96 h) [26]	2	2	2	n.g.	n.g.	0.53	Table 1
cat cornea (transplanted 240 h) [26]	2	2	2	n.g.	n.g.	0.12	Table 1
cat cornea (transplanted 240 h) [26]	2	2	2	n.g.	n.g.	0.45	Table 1
cat cornea (transplanted 240 h) [26]	2	2	2	n.g.	n.g.	0.27	Table 1
human cornea (eye-bank) [48]	2	3	2	58.75	42.6	1.38	Table 1
rabbit lens (magnesium 10 mM 24 h) [14]	3	3	2	59.1	39.1	1.45	Table 1
rabbit lens (magnesium 20 mM 24 h) [14]	3	3	2	33.3	66.7	0.50	Table 1
cat cornea (Optisol) [49]	2	2	2	28.5	71.4	0.40	Table 1
cat cornea (Optisol + hEGF) [49]	2	2	2	29.9	70.1	0.43	Table 1
cat cornea (Optisol + insulin) [49]	2	2	2	24.8	75.1	0.33	Table 1
cat cornea (Optisol hEGF + insulin) [49]	2	2	2	33.9	66.1	0.51	Table 1
rat heart (perfused 5 ppm Cd) [38]	3	2	2	50.7	10.3	4.92	Table 4
rat heart (perfused Cd/Pb) [39]	3	2	2	68.8	15.5	4.44	Table 2
gerbil brain [42]	2	3	2	46.61	53.39	0.87	Table 1
gerbil brain (incub) [42]	2	3	2	17.8	82.1	0.22	Table 1
guinea pig brain incub) [42]	2	3	2	28.04	71.96	0.39	Table 1

Key: Species tissues or organs^Reference^; Nature of gross sample: 2, tissues; 3, organs; Preparation: 2, ex vivo; 3, perchloric acid (PCA) extract; Physiological state: 2, stressed; high-energy (relative) amplitude; low-energy (relative) amplitude; ^31^P spectral modulus; Source, table. n.g., not given.

**Table 3:** ^31^P spectral moduli calculated by dividing the high-energy phosphates by the low-energy phosphates from diseased cells and tissues and examining them using phosphorus-31 nuclear magnetic resonance (^31^P NMR).

Species (Tissues or Organ) ^Reference^	Nature of Gross Sample: (1, Cells; 2, Tissues)	Preparation: (2, Ex Vivo; 3, PCA)	Physiological State: (3, Diseased)	High-Energy Amplitude (Relative)	Low-Energy Amplitude (Relative)	^31^P Spectral Modulus (High-Energy)/(Low-Energy)	Source
chicken pectoralis (dystrophic) [36]	2	2	3	19.1	24.2	0.79	Table 1
human quadriceps, (nemaline rod) [37]	2	2	3	17.3	4.0	4.3	Figure 7B
mouse neuroblastoma [cell lines C-46 (C+)] [50]	1	3	3	18.5	43.8	0.42	Table 1
mouse neuroblastoma [cell lines C-46 (L−)] [50]	1	3	3	21.3	43.8	0.49	Table 1
mouse neuroblastoma [cell linesN-18 (C+)] [50]	1	3	3	12.7	62.8	0.2	Table 1
mouse neuroblastoma [cell lines N-18 (L−)] [50]	1	3	3	6.7	83.9	0.08	Table 1
chicken pectoralis (dystrophic, 10–20 min) [4]	2	2	3	1388	545	2.55	Table 1
human quadriceps (Duchenne) [21]	2	3	3	10.1	11.0	0.92	Table II
human quadriceps (Becker) [21]	2	3	3	12.4	6.8	1.82	Table II
human shoulder (facioscapulohumeral dystrophy) [21]	2	3	3	14.8	14.4	0.91	Table II
human vastus (congenital myotonia) [21]	2	3	3	13.2	14.4	0.92	Table II
human quadriceps (myopathy, etiology unknown) [21]	2	3	3	13.9	13.2	1.05	Table II
human quadriceps (Charcot–Marie–Tooth) [36]	2	3	3	14.3	22.8	0.63	Table II
human gastrocnemius (Kugelberg–Welander) [36]	2	3	3	18.0	23.7	0.76	Table II
human soleus (meningo-myelocele) [36]	2	3	3	17.5	26.4	0.66	Table II
human soleus (cerebral palsy) [36]	2	3	3	11.8	16.4	0.72	Table II
human vastus (amyotrophy after encephalitis) [36]	2	3	3	10.5	10.7	0.98	Table II
human quadriceps (amyotrophy of unknown etiology) [36]	2	3	3	11.0	12.2	0.90	Table II
human colon (cancer) [45]	2	3	3	27.68	75.62	0.37	Table 1
human tumor (malignant) [46]	2	3	3	51.9	51.69	1.00	Table 1
human cornea (keratoconus) [48]	2	3	3	49.18	51.90	0.95	Table 1
human cornea (Fuchs’ dystrophy) [51]	2	3	3	43.70	56.30	0.78	Table 1
human cornea (bullous keratopathy) [51]	2	3	3	47.4	52.6	0.90	Table 1
human cornea (failed graft) [51]	2	3	3	36.7	63.3	0.58	Table 1

Key: Species cells or tissues^Reference^; Nature of gross sample: 1, cells; 2, tissue; Preparation: 2, ex vivo; 3, perchloric acid (PCA) extract; Physiological state: 3, diseased; high-energy (relative) amplitude; low-energy (relative) amplitude; ^31^P spectral modulus; Source, figure/table.

## 3. Results

Cells, tissues, and organs among a total of 98 cases were partitioned into three groups according to their physiologic status: healthy (normal) (Table 1), stressed (Table 2), and diseased (Table 3). These analyses included a group of 11 vertebrate species: bovine, cat, chicken, frog, gerbil, guinea pig, human, porcine, rabbit, rat, and toad. The PSM values calculated from 43 healthy (normal) system cases, 31 metabolically stressed system cases, and 24 diseased system cases averaged, respectively, 1.69 ± 1.11, 0.9 ± 1.07, and 0.99 ± 0.86 (Table 4). The mean modulus value of Table 1 (normal) was greater and significantly different from that of Table 2 (stressed) and Table 3 (diseased). Table 2 and Table 3 were not significantly different from each other.

The variability in the measurements from case to case occurs because the data are derived from different source animal cells, tissues, and organs and additionally derived from different experiments. In support of the case measurements presented, where possible, in each of the published data sets, the standard deviations have been calculated and presented in the references cited.

Considering outliers in healthy (normal) cases, only two cases (4.7%) deviated by two and three standard deviations from the mean (Table 1). In stressed cases, only two cases (6.5%) deviated by two and three standard deviations from the mean, respectively (Table 2). In diseased cases, only one case (4.2%) deviated by three standard deviations from the mean. These outliers were included in the computation (Table 4) of the means for each table.

## 4. Discussion

Considering the number of cases reviewed in compiling Table 1, Table 2 and Table 3, the mean values were tightly grouped. The healthy (normal) value of the mean was greatest, with both the stressed tissues and the diseased tissues being nearly the same and significantly less than normal. This demonstrates that the ^31^P spectral modulus is capable of differentiating between normal and compromised tissues. For normal tissues, the mean value was 1.7; the mean value for stressed tissues was 0.9 and that for diseased tissues was 1.0. Stressed and diseased tissues were essentially the same at a mean in the area of 0.9. These data demonstrate that the modulus is a potentially useful diagnostic tool. In study protocols requiring strict inclusion and exclusion criteria, one would anticipate an increase in the precision of the determinations. Since the data in Table 2 and Table 3 are so close and both stressed and diseased tissues and organs are less than normal, we show herein the potential usefulness of this relatively simple determination.

The ^31^P spectral modulus (PSM) is useful in determining or monitoring the metabolic status of cell, tissue, or organ systems. The PSM can be employed as a calculable numeric assay [23] at a particular time-point or over a time-course, such as in disease development or resolution and provides stress- and disease-dependent information. This has been demonstrated in studies of healthy (Table 1), stressed (Table 2), and diseased (Table 3) tissues and organ systems, or in monitoring the staging of disease treatment regimens or age-related changes using NMR surface coil technology. Since it has been shown that a PCA extract accurately reflects the phosphatic NMR profile of living tissue [3,5,25], in this way ^31^P NMR may be used as a laboratory test. Employing NMR surface coil technology and the PSM in vivo permits the avoidance of the introduction of uncontrolled environmental factors. These factors may include the effects of temperature, humidity, preservation medium, etc. When cells, tissues, and organs are not examined in vivo, the investigator/clinician must be cognizant concerning methods of sample collection and sample treatment.

Data outliers may provide clues regarding the excision or handling of tissues or organs. For example, if a biopsy or tested tissue has little ATP, it might be concluded that the tissues or organs were not handled or prepared optimally. When examining the biopsy/tissue in order to determine if a cell, tissue, or organ is compromised, often, there are tests for the presence of, for example, normal levels of enzymatic activity.

Among all three of the sample groups studied, the most obvious PSM outliers calculated were studies involving the perfused heart organ, where the PSM was considerably higher. The process of perfusing this organ may or may not be considered stressed. Never-the-less, we have assigned these studies to the category of stressed tissues or organs (Table 2), because the heart had been removed from the source animal. However, since ATP contributes the greatest portion to the modulus in the normal [6] and in the stressed or diseased heart [38,39], this high modulus may be expected. The rat heart organ, an example outlier, can be excised from the thorax, cannulated, perfused, and stimulated to resume beating inside a small-bore 15 mm diameter NMR instrument sample tube [38,52]. In this instance, the rat heart has technically an ex vivo status; however, it might even be assumed as having an “in vivo” status, because it performs normally. The beating heart can function completely independently because its function relies on the physiologic buffer, temperature, and nutritional transudate. Neurologically, the heart’s activity functions via the atrioventricular node timer for the heart. Even though there is no neurological connection of the perfused heart with the central nervous system, the atrioventricular node is able to maintain the clocking of the heart. The foregoing raises the question as to the heart’s preparation status. However, upon the excision of the heart from the rat’s thorax, by strict definition, the heart organ’s status is ex vivo. A similar situation would exist for any continuously perfused tissue or organ.

The sampling technique must be recognized, and consideration must be given to it, as it is extremely important in these kinds of cases, and such factors are rarely discussed in the literature. Moreover, it is not often that we have the opportunity to examine so many cases from one laboratory, which can limit the variations that might influence the data herein described. This is the principal reason we restricted this study to the evaluation of cases from our laboratory.

## 5. Study Limitations

The limited number of cases presented herein, albeit nearly 100 derived from our studies, permitted calculable PSM groups for statistical analysis. This restriction was employed in order to control for differences in techniques and methods of preparation of cells, tissues, and organs among laboratories, such as those we detected, described, and discussed in our previous study [6]. Such differences included variations introduced from NMR instrumental specifications, such as the quality, stability, or strength of the magnet and its ability to detect and measure even a single tissue sample, e.g., a whole intact cornea [34].

Other limitations are tissue- or sample-specific variations. For example, the difficulties encountered in procurement of human eye bank corneal tissues, including time of death, time of procurement, type of preservation media, and duration of preservation time. Any or all of these variable conditions can introduce metabolic abnormalities. We were unable to differentiate the effects of these factors on computed moduli determined using corneal tissue. Although obtaining corneal tissues in animal models under controlled conditions is possible, in cases of procuring human corneas, e.g., eye-banked corneas, this presents a particular difficulty in that procurement, e.g., the time of death to procurement, the conditions under which the corneas are excised and immersed in preservation media, and the latter’s chemical composition, is impossible to control.

An important biological observation, as illustrated in Table 1, is the usefulness of the mean value that can still be calculated with very little variance. This implies that energy metabolism in the cases reported is not affected. This is a very profound biological point. Although the crystalline lens of the eye and the beating rat heart muscle are grossly different tissues both physically and metabolically, their moduli are similar. The heart is well vascularized and highly aerobic, whereas the lens is far less aerobic. The organ characteristics of these two vary metabolically, which is further supported by the more acidic lens (pH 6.9) [5]. Its acidity is a consequence of the less aerobic lens metabolism in contrast to the heart (pH 7.2) [39,53]. In lens, the hydrogen ion concentration can be removed and does not accumulate. In contrast, both heart muscle and lens have essentially the same PSM index.

## 6. Future Studies

In an ongoing study, we are currently employing integral analyses calculated using the UN-SCAN-IT Graph Digitizer Software (Silk Scientific, Inc., Provo, UT, USA). This permitted analyses of a large number of published spectra incorporating published data from phylogenetically diverse samples from all realms of phylogeny. Using surface coil technology, we can apply calculation of the PSM to the study of the physiological state of stressed and diseased systems, whether they arise from infections or from metabolic, inflammatory, or genetic disease, aging, or the influence of treatment protocols.

## 7. Conclusions

The establishment of ATP as a hydrotrope supports a fundamental change in a long-held hypothesis that the main or principal function of ATP was as cellular currency [6,9,10]. In healthy cells, tissues, and organs, ATP is also the organophosphate with the highest intracellular concentration. It is the principal phosphate of the ^31^P high-energy-phosphate band of the NMR spectrum, whereas inorganic orthophosphate and sugar phosphates are constituents of metabolic processes. ATP functioning in the prevention of protein aggregation or maintenance of protein solubility is foundational. As such, it can prevent cellular, tissue, and organ dysfunction, as well as enzyme and perhaps structural failure that can be measured in vivo, ex vivo, and in vitro and by the analysis of extractions with the ^31^P NMR spectral modulus. The calculation of the PSM does not require highly resolved NMR spectral signals but relies on the minimal resolution required to detect an integral curve with a clear spectral inflection point just downfield of the phosphocreatine signal (−3 δ) [18]. The detection of an integral curve alone is now possible and has been reduced to practice using data from living tissues already established by surface-coil technology [23]. The calculation of a PSM is possible even at levels of phosphorus concentration so low as to prevent detection of the individual or groups of metabolites, such as in vivo or ex vivo cells, tissue, or organ determinations. With increasing stress or maturation of disease in cell, tissue, or organ systems, the PSM index declines; alternatively with decreasing stress or resolution of disease states, the PSM increases. Determination of the PSM using presently deployed surface-coil technology allows for the determination of homeostasis, health status, and monitoring changes in vivo associated with metabolic decline, as in senescence, and pathology in cell, tissue, and organ systems or even endeavors into metabolic enhancement in compromised in vivo systems.

## Figures and Tables

**Table 4:** Pair-wise comparisons of compiled ^31^P spectral moduli, Table 1, Table 2 and Table 3, by T-TEST.

Table	N	Mean	Std. Dev.	Probability (Tables)		
				1 with 2	1 with 3	2 with 3
1, normal	43	1.694	1.109	0.003 *		
2, stressed	31	0.901	1.070		0.005 *	
3, diseased	24	0.987	0.863			0.743

* Significant at *p* < 0.05.

## Data Availability

The data presented in this study are available in the main article.
